# How We Can Reap the Full Benefit of Teleconsultations: Economic Evaluation Combined With a Performance Evaluation Through a Discrete-Event Simulation

**DOI:** 10.2196/32002

**Published:** 2022-05-20

**Authors:** Marius Huguet, Marianne Sarazin, Lionel Perrier, Vincent Augusto

**Affiliations:** 1 Mines Saint-Etienne, Univ Clermont Auvergne, CNRS, UMR 6158 LIMOS, Centre CIS F-42023 Saint-Étienne France; 2 Groupe mutualiste Aesio F-42100 Saint-Étienne France; 3 IPLESP- Umrs 1136 Inserm, réseau Sentinelles F-75012 Paris France; 4 Univ Lyon, Leon Berard Cancer Centre, GATE UMR 5824 F-69008 Lyon France; 5 Human and Social Science Department, Centre Léon Bérard F-69008 Lyon France

**Keywords:** telemedicine, telehealth, teleconsultation, discrete-event simulation, economic evaluation, propensity score matching

## Abstract

**Background:**

In recent years, the rapid development of information and communications technology enabled by innovations in videoconferencing solutions and the emergence of connected medical devices has contributed to expanding the scope of application and expediting the development of telemedicine.

**Objective:**

This study evaluates the use of teleconsultations (TCs) for specialist consultations at hospitals in terms of costs, resource consumption, and patient travel time. The key feature of our evaluation framework is the combination of an economic evaluation through a cost analysis and a performance evaluation through a discrete-event simulation (DES) approach.

**Methods:**

Three data sets were used to obtain detailed information on the characteristics of patients, characteristics of patients’ residential locations, and usage of telehealth stations. A total of 532 patients who received at least one TC and 18,559 patients who received solely physical consultations (CSs) were included in the initial sample. The TC patients were recruited during a 7-month period (ie, 2020 data) versus 19 months for the CS patients (ie, 2019 and 2020 data). A propensity score matching procedure was applied in the economic evaluation. To identify the best scenarios for reaping the full benefits of TCs, various scenarios depicting different population types and deployment strategies were explored in the DES model. Associated break-even levels were calculated.

**Results:**

The results of the cost evaluation reveal a higher cost for the TC group, mainly induced by higher volumes of (tele)consultations per patient and the substantial initial investment required for TC equipment. On average, the total cost per patient over 298 days of follow-up was €356.37 (US $392) per TC patient and €305.18 (US $336) per CS patient. However, the incremental cost of TCs was not statistically significant: €356.37 – €305.18 = €51.19 or US $392 – US $336 = US $56 (95% CI –35.99 to 114.25; *P*=.18). Sensitivity analysis suggested heterogeneous economic profitability levels within subpopulations and based on the intensity of use of TC solutions. In fact, the DES model results show that TCs could be a cost-saving strategy in some cases, depending on population characteristics, the amortization speed of telehealth equipment, and the locations of telehealth stations.

**Conclusions:**

The use of TCs has the potential to lead to a major organizational change in the health care system in the near future. Nevertheless, TC performance is strongly related to the context and deployment strategy involved.

## Introduction

In recent years, the rapid development of information and communications technology (ICT) has contributed to expanding the scope of application and expediting the development of telemedicine [1]. Initially, telemedicine was mainly developed to tackle medical desertification in remote areas and as a useful tool in the context of natural and human-made disasters [2-4]. The primary objective of telemedicine was to enhance patients’ access to care by offering the opportunity for patients located in remote areas to gain fast and distant access to medical expertise through the use of ICT [3]. More recently, the COVID-19 crisis has been a turning point in the development of telemedicine and has drastically accelerated its adoption. The use of telemedicine has soared, with 486,369 teleconsultations (TCs) performed in France during the last week of March 2020, against an average of 10,000 TCs per week before the COVID-19 crisis [5].

Telemedicine is a generic term gathering several heterogeneous subcategories, such as TC, telemonitoring, teleassistance, and tele-expertise [6]. Focusing on TCs, one can further distinguish between video TC and TC using a telehealth station [7]. The former refers to a TC involving a patient and a distant physician using a videoconferencing solution. Videoconferencing solutions dedicated to TCs are software programs that include various types of services. The latter refers to a TC involving, on the one hand, a patient accompanied by a medical professional and, on the other hand, a distant physician. A telehealth station is a piece of hardware that includes a videoconferencing solution and a variety of connected medical devices (eg, a stethoscope, a handheld camera, an ultrasound scanner, an electrocardiogram). The connected medical devices are handled by the medical professional who is accompanying the patient, and they allow real-time transmission of data to the distant physician for interpretation. In France, many telehealth stations have been installed in nursing homes or in drug stores. This process has been facilitated by rider 6 to the French agreement for nurses, which recognized accompaniment by nurses as a medical act with an associated fee per TC [8]. Similarly, rider 15 to the French convention of pharmacists sets a flat-rate remuneration based on the annual volume of TCs completed [9].

In the scientific literature, the use of TCs has been intensively studied from a variety of perspectives and in a variety of contexts. Many studies have questioned the relative quality of care of a TC compared with a physical consultation (CS) by conducting randomized controlled trials. A few of these studies have used generic health measures such as quality-adjusted life years [10,11]. Nevertheless, TCs were found to have no impact on such long-term patient outcomes. To increase the likelihood of detecting small changes in quality of care, other studies have relied on various disease-specific measures (eg, diagnostic accuracy, reduction in wound size, blood glucose level) [11-13]. Overall, these studies have tended to demonstrate that the use of TCs is a safe alternative to CSs, providing a noninferior level of quality in a variety of contexts. The question of quality of care has also been investigated from the patient perspective through questionnaires concerning satisfaction and patient-reported outcomes. A recent randomized controlled trial on orthopedic consultations based on the 3-level version of the EuroQol 5-dimensional system (EQ-5D-3L) found no difference in perceived quality of care [14]. Regarding patient satisfaction with TCs, several surveys have been conducted (ie, both disease specific or nondisease specific), indicating a high level of satisfaction [15-17].

Many economic evaluations have also been conducted through cost analysis or cost-effectiveness analysis. Five reviews of the existing literature on economic evaluations addressing studies prior to 2010 failed to reach any reliable conclusion, arguing that economic evaluations of telemedicine were less adherent to methodological standards than evaluations in other fields (eg, featuring a lack of information on the costing methodology, the perspective of the evaluation, and sensitivity analysis) [6,18-21]. In a recent literature review focusing on telemonitoring, the French Authority for Health observed a substantial increase in the methodological quality of evaluations compared with a previous review conducted by the Authority in 2013 [6,22]. More recently, 2 literature reviews addressed studies published during the period 2014-2020. One of these was a scoping review that included 50 economic evaluations of telemedicine [23], which was found to result in cost savings of 53%, 50%, and 32% in cost-minimization, cost-effectiveness, and cost-utility analyses, respectively. These analyses tended to identify increased productivity through a reduction in consultation time; however, such a reduction might be offset by the associated increase in administrative overhead. Furthermore, a reduction in resource consumption is unlikely to result in cost savings under an activity-based payment scheme. The scoping review substantiated several scenarios in which telemedicine could lead to cost savings, such as when medical patient transportation could be avoided. The other literature review was an umbrella review that included 18 systematic reviews on costs or cost-effectiveness analysis [24]. Among the 18 systematic reviews included, 7 concluded that telemedicine was cost saving, 4 concluded that telemedicine was more expensive, and the remaining 7 reviews were unable to reach a conclusion due to heterogeneity in the outcome measures and the poor quality of the cost data. Overall, the heterogeneity in the conclusions among the studies included in these 2 literature reviews may partially be explained by the variety of diseases and contexts in which telemedicine was evaluated. Moreover, the multitude of diseases investigated independently in a disease-specific setting prevented many studies from considering the amortization of telemedicine equipment as the rate of equipment utilization for other diseases was unknown.

In this context, the aim of this study is twofold. First, this study intends to provide an economic evaluation of the use of TCs for specialist consultation at hospitals through a cost analysis. Second, we conduct a performance evaluation based on a discrete-event simulation (DES) with key performance indicators (KPIs) such as costs, travel time, and resource time consumption.

In fact, several studies have employed simulation and modeling techniques to assess the performance and facilitate the deployment of telemedicine in a variety of contexts. Modeling techniques such as Petri nets have been used to describe health care systems formally with the purpose of performance evaluation. A generic modeling approach to alarm management workflows in health care was proposed by Fanti et al [25] using UML (uniform modeling language) for communication and colored timed Petri nets for simulation. The framework was shown to have high potential capability for describing large and complex health care systems. Dotoli et al [26] proposed a continuous Petri net framework to describe the structure and dynamics of an emergency cardiology department. In the same paper, the model allowed for the generation of an optimization problem and a simulation model. Dotoli et al [27] also used UML activity diagrams and Petri nets to improve the management of hospital departments, and proposed a case study on a pulmonary department. The authors claimed that their base model could be used to design and size any hospital department. Hamana et al [28] used Petri nets to model patient care pathways along with information flows. The authors proposed a performance evaluation approach through Petri net simulation, taking into account degraded modes related to information communication problems.

Regarding simulation, such an approach was used to facilitate the deployment of a telemedicine program in Mexico that consisted of a mobile unit aimed at providing TCs with a distant expert to people in extreme poverty or remote locations [29]. Based on a DES, the authors built a flexible model to calibrate the resources (eg, physicians, mobile units, satellite coverage) to increase the program’s utilization rate. Similarly, Qiao et al [30] proposed a DES model to calibrate the resources (eg, TC rooms at hospitals and physicians) to minimize patient waiting times. Considering empirical TC flows from the Henan Telemedicine Center of China, they proposed an optimal sizing of the resources for that hospital. From a theoretical perspective, another study explored the optimal allocation of resources (eg, TC rooms, experts) that would minimize waiting times in the provision of TCs [31]. Based on queuing theory, the findings of the study indicated that the combination of the number of experts and TC rooms does indeed have a decisive impact on the queue length and that the impact of TC rooms is much larger. In a different context, a study investigated the use of TCs as a way for specialists to review patient referrals to remove inappropriate patients from specialist queues [32]. Using a DES approach and data from a rheumatology clinic, the authors found that without TCs, lead times were very long, and the use of TCs as a triage tool was found to be very effective in increasing the performance of the system. Based on a French experiment on telemedicine in geriatrics launched in 2006, a study employed a system dynamics approach through a parametric scenario–based model to compare the performance of a system without telemedicine with 3 alternative scenarios involving the use of telemedicine (eg, TCs only, tele-expertise only, and both TCs and tele-expertise) [33]. Assuming that quality of care with telemedicine and quality of care without telemedicine are comparable, they considered KPIs such as total health care costs, carbon dioxide equivalent emitted, and total medical time available. Their findings favored the tele-expertise scenario for increasing the total medical time available, while the scenario combining tele-expertise and TCs tended to be superior in terms of total costs and environmental aspects.

The uniqueness of our study lies in part in the combination of an economic evaluation of TCs through a cost evaluation and a performance evaluation that uses a DES approach. The cost analysis seeks to determine whether the way in which TCs were deployed at a much larger scale during the early stages of the COVID-19 pandemic (ie, early 2020) was a cost-saving strategy, and the DES approach explores the performance associated with several alternative scenarios for future deployment. The DES approach also substantiates the importance of resources other than cost in the evaluation, such as medical time and administrative time. Our evaluation framework is not disease specific and, instead, considers any eligible specialty. Our study thus contributes to the literature on economic evaluations by investigating in more detail the issue of telemedicine equipment amortization by taking into account all specialties that might benefit from investments in this equipment. Moreover, we compare patients’ care pathways over a 298-day period of follow-up in the economic evaluation, which allows us to shed light on changes in the demand for (tele)consultations. Furthermore, to the best of our knowledge, our study is the first to distinguish between TCs using a videoconferencing solution and TCs using a telehealth station. This distinction allows us to investigate various scenarios for the future deployment of TCs based on the relative intensity of use of CSs, video TCs, and TCs using a telehealth station within the eligible population. However, we are able to make this distinction only in the DES approach; in the economic evaluation, video TCs and TCs using a telehealth station are merged into a single group as it was not possible to obtain the relevant information at the individual level in the data. Our approach substantially differs from that of studies investigating the best allocation of resources given a flow of TCs because we evaluate the performance associated with various levels of intensity of TC use (ie, which lead to different TC flows) given a population.

## Methods

### Positioning of the Problem

**Figure 1:**
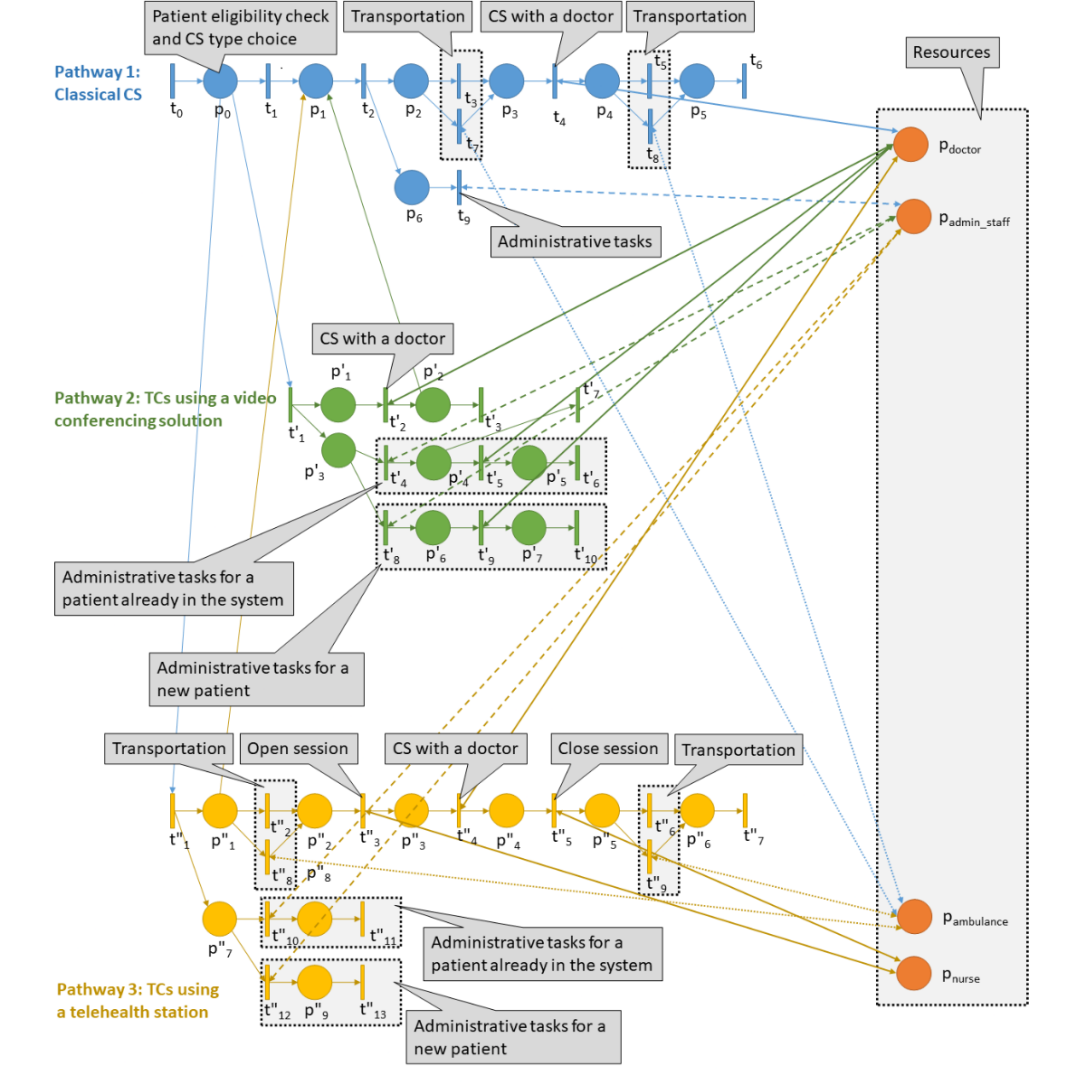
Petri net of patient pathways. CS: physical consultation; TC: teleconsultation.

In this paper, we propose a generic model of TC use, taking into account 3 alternatives: (1) classical CSs (pathway 1), (2) TCs using a videoconferencing solution (pathway 2), and (3) TCs using a telehealth station (pathway 3). To formally define the corresponding patient pathways, we propose a Petri net model illustrated in Figure 1. Pathways 1, 2, and 3 are represented using a different color: blue, green, and yellow, respectively.

Source transition t_0_ models the arrival of consultation requests in the system. Place p_0_ models the choice between classical CSs (the patient is not eligible for a TC, transition t_1_), TCs using a videoconferencing solution (transition t′_1_), and TCs using a telehealth station (transition t″_1_). Places p_doctor_, p_admin_staff_, p_ambulance_, and p_nurse_ model the resources taken into account.

In pathway 1, transition t_2_ models the activation of 2 parallel activities related to the consultation itself (pathway p_2_-t_6_) and administrative work (pathway p_6_-t_9_). The patient goes to the CS using his/her personal vehicle (transitions t_3_ and t_5_) or an ambulance (transitions t_7_ and t_8_). Transition t_4_ models the CS with a doctor. The patient then exits the system (sink transition t_6_). Finally, transition t_9_ models administrative tasks executed in the background.

In pathway 2, transition t′_1_ models the activation of 2 parallel activities related to the consultation itself (p′_1_-t′_3_) and administrative work (p′_3_-t′_10_). The video TC is performed with a doctor (transition t′_2_). Then, the patient may need a classical CS and be redirected to place p_1_ in pathway 1. Otherwise, the patient exits the system (sink transition t′_3_). If the patient is new (pathway p′_3_-t′_7_), the administrative tasks and consultation are longer than the pathway (t′_8_-t′_10_).

In pathway 3, transition t″_1_ models the activation of 2 parallel activities related to the consultation itself (pathway p″_1_-t″_7_) and administrative work (pathway p″_7_-t″_13_). If the nurse is sick (place p″_1_), the patient is redirected to a classical CS. Otherwise, the patient goes to the equipment location using a personal vehicle (transitions t″_2_ and t″_6_) or an ambulance (transitions t″_8_ and t″_9_). Then, the nurse sets up the equipment and opens the session (transition t″_3_), the doctor performs the consultation (transition t″_4_), and the nurse closes the session (transition t″_5_). The patient then exits the system (sink transition t″_7_). Finally, depending on whether the patient is new (t″_10_-t″_11_) or not (t″_12_-t″_13_), administrative tasks with different durations are performed.

Based on this formal model, the problem addressed in this paper consists of proposing control policies to optimize the operations of the global system. Such control policies are strongly connected to decision p_0_, that is, how to assign a pathway to a patient taking into account his/her characteristics. Traveling durations and the amount of available resources (doctors, administrative staff, ambulances, and nurses) are also taken into account. The proposed model followed the recommendation of the French Authority for Health to capture organizational changes induced by health technologies, for example, by considering changes in processes (eg, the appointment process, consultation process) induced by the use of TCs [34].

### Data

In this study, we used 3 data sets to obtain detailed information on the characteristics of patients, the characteristics of patients’ residential locations, and the usage of telehealth stations. First, we identified all patients who had at least one CS or TC with a specialist at the *Clinique Mutualiste de Saint-Etienne* private hospital from January 2019 to September 2020. This clinic specializes in surgeries related to fields such as urology, orthopedics, digestive medicine, gynecology, and treatment of obesity, with 15% of its practice dedicated to cancer. The clinic also has a geriatric department and a dementia center. From January 2019 to March 2020 (the beginning of the COVID-19 pandemic in France), telemedicine was not part of Clinique Mutualiste medical practice. As the pandemic increased in severity, all French hospitals were forced to limit care to patients with COVID-19 or urgent illness. Therefore, all surgical interventions and care for low emergency cases were postponed. To stay in touch with patients and to ensure psychological follow-up, especially if the patients had cancer, obesity problems, or dementia or needed anesthetic advice before a surgery that cannot be postponed, Clinique Mutualiste established a telemedicine program. This program consisted of 2 telemedicine cabins with competent nurses outside the clinic for patients who needed a total examination and special visual software that could be downloaded by patients on their phone or computer. All practitioners working in all medical specialties were taken into account. Other telehealth stations were also introduced in 10 nursing homes around the clinic within the Loire department.

The inclusion criteria were having at least one (tele)consultation during the period of follow-up and that the (tele)consultations were performed before or after a hospital stay. The link with a hospital stay is required to obtain information on patient characteristics from patient records. We relied on a fixed period of follow-up to have the same chance of observing a (tele)consultation for each patient. To maximize the number of TCs included in the sample, we retained the period of follow-up of 165 days before hospitalization and 133 days after hospitalization (see Multimedia Appendix 1 for more details). For each patient, the information recorded included the residential location of the patient, his/her age, gender, ICD-10 (International Statistical Classification of Disease and Related Health Problems, tenth revision) diagnostic codes (see Multimedia Appendix 2 for more details), care unit (eg, ambulatory, gynecology, urology, neurology), and date of discharge as well as the date of each (tele)consultation. We also computed the travel distance and travel time by car between patients’ residential municipalities and the private hospital.

Second, we included aggregate information about patients’ residential locations from open access data sets managed by the *National Institute of Statistics and Economic Studies* (INSEE). We obtained information about the municipalities, such as the population density and median standard of living (in euros) within each municipality in mainland France.

Finally, we included data on the usage of telehealth stations in the department of Loire from an application programming interface (API) provided by HOPI Medical. The information recorded included the date of each TC performed using a telehealth station and the TC time (minutes).

### Ethics Approval

This study was conducted in accordance with the ethical principles for medical research involving human subjects developed in the Declaration of Helsinki by the World Medical Association (WMA). The study received approval in France from the National Ethics Committee (CESREES N°2809078 bis, CNIL N°921041).

### Economic Evaluation (Cost Analysis)

We first conducted a cost analysis of the use of TCs versus CSs over 298 days of follow-up (see Multimedia Appendix 1). The analysis was conducted from a health care system perspective. In fact, using a retrospective data set, it was not possible to retrieve data on informal care, which prevented us from considering a collective perspective. Based on the recommendations of the French Authority for Health, costs were not discounted since the time horizon was less than 12 months [35].

The 2 strategies being compared are as follows:

- TC patients: patients having at least one TC during the follow-up period. TCs performed using a videoconferencing solution or a telehealth station were merged in the same group as it was not possible to distinguish between the 2 types of TCs at the individual level in the data.
- CS patients (the control group): patients having solely CSs during the follow-up period.

An important feature of the economic evaluation is that we evaluated the cost differences over patients’ care pathways (ie, 298 days of follow-up). Therefore, a TC patient might also have had CSs during his/her care pathway, and these CSs are taken into account in the total cost. By doing so, we evaluated a mixed organization of care in which patients might be treated with both TCs and CSs during their care pathway. The incremental cost between our 2 groups of interest is computed. Based on the recommendations of the French Authority for Health, we considered direct production costs only (ie, costs of the resources required for the production of the interventions evaluated). All costs are valued in euros (2020 data). The total cost for patient *i* is computed as follows based on the cost inputs presented in Table 1:



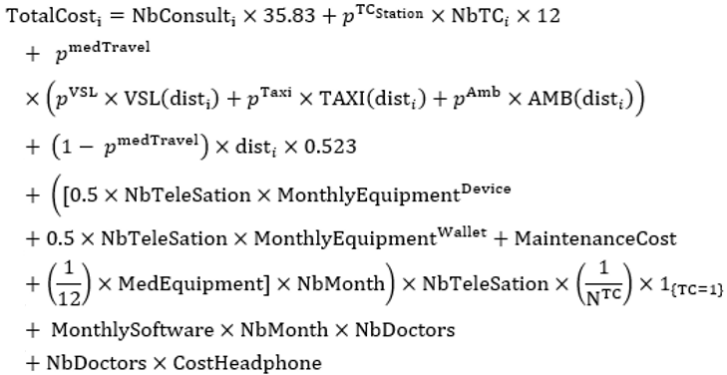



where NbConsult*_i_* is the number of (tele)consultations (all specialties) during the period of follow-up; *p*^TC Station^ is the probability that a TC was performed using a telehealth station; NbTC*_i_* is the number of TCs (all specialties) during the period of follow-up; *p*^medTravel^ is the share of medical patient transportation; *p*^VSL^, *p*^Taxi^, and *p*^Amb^ are the probability of using a light health vehicle (VSL), a taxi, or an ambulance, respectively, if a patient uses medical transportation; dist*_i_* is the cumulative distance traveled by patient *i* during the period of follow-up; VSL(), TAXI(), and AMB() are the functions returning the cost of transportation by a VSL, a taxi, and an ambulance, respectively, for a specified distance (see Multimedia Appendix 3 for more details); NbTeleStation is the number of telehealth stations; MonthlyEquipment^Device^ includes the cost of a full telehealth station, cost of installation, cost of consumable medical equipment, and cost of medical staff training. To take into account the period of usage and the depreciation of the TC equipment, this cost is converted into a monthly cost over the entire period of usage (eg, 60 months if full depreciation at 5 years is assumed); MonthlyEquipment^Wallet^ includes the cost of a telehealth station (wallet), cost of consumable medical equipment, and cost of medical staff training; MaintenanceCost is the cost of maintenance of a telehealth station (monthly); MedEquipment is the medical equipment per telehealth station; NbMonth is the number of months (time window) of the study; N^TC^ is the number of patients who had at least one TC. The frequency was computed before any matching procedure or censoring procedure (ie, period of follow-up) to take into account the real/observed TC usage during the study period; NbDoctors is the number of doctors trained to use TCs within the hospital; MonthlySoftware is the cost of the videoconferencing solution (per month and per user); and CostHeadphone is the cost of a headphone and camera per user.

**Table 1:** Main cost inputs and other inputs (in euros [2020 data])^a^.

Inputs		Value	Sources
**TC^b^ station equipment costs**			Financial data, private hospital in Saint-Etienne
	Telehealth station, full station (per station)	21,686.4	
	Telehealth station, wallet (per station)	10,200	
	Medical equipment (per station)	360	
	Installation cost (per station)	1680	
	Training (per team of 4 medical professionals)	1020	
	Maintenance (per month per station)	90	
	Consumable medical equipment (per year or per 200 TCs)	360	
**Video TC equipment costs**			Financial data, private hospital in Saint-Etienne
	Software and storage server (per month per user)	70	
	Camera and headphone (per user)	110	
**Patient transportation costs**			
	Nonmedical patient transportation (cost/kilometer)	0.523	[36]
	Medical patient transportation	See Multimedia Appendix 3	Multimedia Appendix 3
**(Tele)consultation costs (per consultation)**			
	Average cost for a specialist (tele)consultation (including average out-of-pocket fees)^a^	35.83	[37]
	Tariff for nurse accompaniment during a TC	12	[8]
**Other inputs**			
	Share of medical patient transportation	0.36	Assumption
	Number of telehealth stations	12	Data, nursing homes in the department of Loire (Aésio group)
	Share of telehealth stations with connected devices	0.5	Data, nursing homes in the department of Loire (Aésio group)
	Number of teams of 4 medical professionals per telehealth station	1	Data, nursing homes in the department of Loire (Aésio group)
	Depreciation rate of TC equipment	5 years	Assumption
	Probability that a TC was performed using a telehealth station (*P*^TC Station^)	0.2656	Data, HOPI Medical
	Number of doctors using TCs	30	Data, private hospital in Saint-Etienne

^a^Costs were reported in 2017 euros in the report from the French National Health Insurance, and were expressed in 2020 euros using the international Classification of Individual Consumption by Purpose (COICOP 06.2.1.2.1) discount rate. €1 = US $1.1 (2020 data).

^b^TC: teleconsultation.

The cost evaluation for CS patients includes the average cost for a specialist CS (including average out-of-pocket fees) multiplied by the number of CSs observed during the period of follow-up as well as the cost of patient transportation. We computed the average cost for a specialist CS, including average out-of-pocket fees, based on open access data for 2017 taken from the French National Health Insurance report [37]. Since the latest data set available was from 2017, the average cost in 2020 euros was expressed using the COICOP 06.2.1.2.1 discount rate. Nonmedical transportation was valued using the official cost per kilometer according to French legislation [36]. Medical transportation was valued for each transportation mode (eg, VSL, Taxi, Ambulance) according to the conventional tariffs set by the French National Health Insurance [38-40]. Then, the average medical transportation cost was computed as the sum of each transportation cost weighted by its probability of use. The probability of use of each transportation mode was derived from a report by the French Directorate for Research, Studies, Assessment, and Statistics (DREES) based on 2018 health expenses data [41]. Finally, transportation costs were computed as the sum of medical and nonmedical transportation costs weighted by the assumed share of patients using medical transportation. For TC patients, the cost evaluation further included TC equipment costs, the training of medical staff, and the fee for nurse accompaniment in cases where a telehealth station was used. Cost inputs were retrieved from financial data taken from the private hospital in Saint-Etienne (France). To overcome the problem posed by the fact that we could not distinguish between the 2 types of TCs (ie, video versus telehealth station) in the data, the tariff for nurse accompaniment was weighted with the probability that a TC was performed using a telehealth station. While we do not observe the type of TC (ie, video versus telehealth station) at the individual level, data from HOPI Medical allow us to compute the share of TCs performed using a telehealth station. The investment in TC equipment is assumed to be fully depreciated in 5 years, and we assume in the economic evaluation a constant rate of utilization of TC equipment during that period. Additionally, the cost of TC equipment is spread over the real number of patients who had a TC before any exclusion criteria were applied.

In the comparison of costs between our 2 groups of interest, selection bias is likely to occur, induced by the fact that not all patients are eligible for the use of TCs, depending on their individual or disease characteristics. In the economic evaluation, we need to control for this selection bias because the potential differences in characteristics could introduce bias when evaluating the costs. To that end, we rely on a propensity score matching procedure to select a representative control group [42,43]. The propensity score is the conditional probability that a patient will be part of the treatment group (ie, part of the TC patient group), conditional on observable characteristics. This conditional probability is used as a unidimensional indicator of patient characteristics. In other words, 2 patients with a similar propensity score should have similar characteristics (ie, characteristics involved in the estimation of the propensity score). We determined this probability by fitting the following logit model through maximum likelihood estimation:



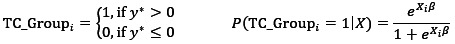



where the latent variable y* = *X*′*β* + ; the parameter vector *β* is obtained through maximum likelihood estimation; and follows a logistic distribution. The matrix of patient characteristics *X*′ includes age, gender, the travel time to the hospital, the median standard of living, population density, the care pathway type, the ICD-10 chapter, and the care unit. The decision regarding which variables to include in the logit model should not be based on their expected predictive power. If the propensity score model was designed as a classifying model and assuming a high accuracy of that model, the matching procedure would fail to balance patient characteristics because the patients in the treatment group would have a probability close to 1 and the patients in the control group would have a probability close to 0. Instead, one should include all variables suspected of inducing selection bias between the 2 groups being compared. We then conducted a 1:1 matching procedure by matching each TC patient to the closest CS patient in terms of the propensity score.

To take into account the uncertainty surrounding the point estimation of the incremental cost, we conducted a probabilistic sensitivity analysis. We used a nonparametric bootstrap procedure with 1000 replications [44]. Nonparametric bootstrapping is a resampling procedure where each bootstrap sample is generated by a random sample with replacement from the initial data set. This method is widely used in statistics to obtain the distribution of a point estimate. We also conducted a deterministic sensitivity analysis to explore the sensitivity of the mean difference in cost to the variations in several cost inputs through a tornado diagram.

### Performance Evaluation (DES)

We implemented the Petri net model presented in Figure 1 using a DES approach. The population was generated by randomly selecting each agent with replacement from the 2020 clinical database described in the “Data” section. The model was simulated over a 5-year period (253 working days per year) to cope with the assumed amortization time of TC equipment. The eligibility criteria for TC patients were based on agents’ attributes (eg, ICD-10 chapters, care units), excluding from the eligible population attributes never observed for TC patients in the data. Finally, conditional on the eligible population, agents were dispatched among the 3 consultation types based on probabilities. Considering that patients older than 80 years old are less likely to be eligible for video TCs, we assumed that for this subpopulation, the probability *P*^visio^ is upper bounded at 0.3. Thus, in scenarios in which *P*^visio^ exceeds 0.3, the residual probability *P*^visio^ – 0.3 is attributed equivalently to *P*^CS^ and *P*^TC Station^. Table 2 provides a full description of the calibration of the parameters.

In the simulation, we assumed a maximum capacity of resources (medical transportation, doctor, nurse, and administrative staff times) and tracked their level of use. For telehealth stations, however, we specified a limited capacity. We assumed that each station could handle a maximum of 7 TCs per day. When the daily workflow exceeds the maximum capacity, the agent is redirected to a CS.

This study is not restricted to a single specialty or disease, and it aims to evaluate the use of TCs from an organizational perspective. Therefore, in accordance with the existing literature (see the “Introduction” section), we assume that the clinical effectiveness of TCs and CSs is comparable among patients eligible for TC [10-14]. This assumption does not negate the fact that not all patients are eligible for the use of TCs, depending on individual or disease characteristics. Thus, we consider the KPIs in Textbox 1 in the performance evaluation of the system.

We conducted 3 experiments and an extra validation experiment (ie, base scenario), which are summarized in Table 3. In experiment 1, we explored the performance of the model associated with all combinations of probabilities *P*^CS^, *P*^visio^, and *P*^TC Station^. These probabilities reflect the intensity of use of each type of consultation, and thus, they allow us to evaluate the performance associated with various degrees of deployment of each TC type. As the use of video TCs and TCs using telehealth stations is subject to substantial initial investments (ie, fixed costs), it is a particularly insightful factor in examining variation in performance based on the volume (ie, probabilities) of each type of TC. This experiment will, for example, highlight the minimum volume of video TCs and TCs using a telehealth station required to amortize the videoconferencing system and telehealth stations, respectively.

**Table 2:** Parameters of the simulation model.

Parameters		Value	Source
Arrival rate (per day)		Poisson(89.953782) in 2020	Clinical data
Probability that a new patient first had a TC^a^ (to be multiplied by 1/number of TCs per patient to convert at the consult level)		0.2368	Clinical data (number of first TC/number of TC patients)
Probability of a first TC (not previously registered by the doctor)		0.8512	Clinical data (1/number of TC per patient)
Probability of having a CS^b^ (*P*^CS^)^c^		0.9507	Clinical data
Probability of having a video TC (*P*^visio^)^d^		0.0362	Clinical data
Probability of having a TC using a telehealth station (*P*^TC Station^)^e^		0.0131	Data, HOPI Medical
**CSs**			
	Travel time (1 way)	Individual agent travel time	Clinical data
	Appointment scheduling time	Triangular(1, 2, 1.5)	Secretary staff
	CS time (doctor)	Triangular(16.9, 29.1, 20)	[45]
**Video TCs**			
	Consultation time (doctor)	Triangular(8, 9.47, 13.78)	Consult time reduced by 52.6% for otorhinolaryngology [15]
	Appointment scheduling time (admin)	Triangular(1, 2, 1.5)	Secretary staff, private hospital in Saint-Etienne
	Appointment scheduling time for new patients (admin)	Triangular(8, 10, 9)	Secretary staff, private hospital in Saint-Etienne
	Registration time for a first TC (doctor)	Triangular(1, 2, 1.5)	Secretary staff, private hospital in Saint-Etienne
	Probability of having a CS within 7 days after a TC	0.0672	Clinical data
**TCs using a telehealth station**			
	Travel time (1 way)	0	Assumption
	Preparation time (nurse)	Triangular(8, 12, 10)	Protocol private hospital
	Consultation time (doctor)	Lognormal(2.1888426, 0.57548749, 3.0333333)	Data HOPI Medical
	Closing time (nurse)	Triangular(3, 7, 5)	Protocol private hospital
	Probability of canceling the TC due to the sick leave of a nurse	0.05/number of teams per telehealth station	Assumption
	Appointment scheduling time (admin)	Triangular(2.5, 4.5, 3.5)	Department of Anesthesia of a private hospital in Saint-Etienne
	Appointment scheduling time for new patients (admin)	Triangular(10, 15, 12.5)	

^a^TC: teleconsultation.

^b^CS: physical consultation.

^c^*P*^CS^: CS probability.

^d^*P*^visio^: video TC probability.

^e^*P*^TC Station^: TC using a telehealth station probability.

**Key performance indicators considered in the performance evaluation of the system.**

**Cost key performance indicator**

When an agent exits the system, we compute the total cost based on the agent’s characteristics (eg, distance traveled) and care pathway based on the same formula used in the economic evaluation. We then sum each total cost over all agents in a simulation run to obtain the overall total cost of the system. Similarly, we compute the average cost associated with each consultation type to obtain the cost per physical consultation (CS), cost per video teleconsultation (TC), and cost per TC using a telehealth station.

**Resource usage key performance indicator**

Includes doctor, administrative staff, and nurse times (minutes). When an agent consumes a resource, we keep track of the level of consumption by taking a random draw from the distributions in Table 2.

**Transfer key performance indicator**

The number of patients transferred to a CS after a video TC or after a TC using a telehealth station due to the unavailability of the station or the sick leave of a nurse.

**Volume of (tele)consultation key performance indicator**

The number of CSs, video TCs, and TCs using a telehealth station completed.

**Travel time key performance indicator**

The total travel time and travel time avoided (minutes). We compute the travel time avoided for each agent engaging in a TC (ie, either using a video TC or via a telehealth station) as the distance he/she would have traveled to the clinic based on his/her characteristics.

**Table 3:** Descriptions of the experiments.

Description			Base scenario		Experiment 1		Experiment 2		Experiment 3
**Simulation features**									
	Replication count	20		20		20		20	
	Replication length (minutes)	1,821,600		1,821,600		1,821,600		1,821,600	
**Parameters variation**									
	*P* ^CSa^	0.9507		Combination summing to one^b^		0 (0.01) 1		Combination summing to one^b^	
	*P* ^visioc^	0.0362		Combination summing to one^b^		0		Combination summing to one^b^	
	*P* ^TC Stationd^	0.0131		Combination summing to one^b^		1 – *P*^CS^		Combination summing to one^b^	
	Share medical transport	0.36		0.36		1		0.36	
	Number of telehealth stations	12		12		12		12	
	Number of teams of nurses per station	1		1		1		1	
**Population variation**									
	Poisson daily arrival rate	89.95		89.95		20.64		89.95	
	Age (years)	No restriction		No restriction		Age >80		No restriction	
	Distance (km)	No restriction		No restriction		13.06		Distance<20; 20≤Distance<50; Distance≥50	

^a^*P*^CS^: physical consultation probability.

^b^*P*^CS^, *P*^visio^, *P*^TC Station^ as long as *P*^CS^ + *P*^visio^ + *P*^TC Station^ = 1.

^c^*P*^visio^: video teleconsultation probability.

^d^*P*^TC Station^: teleconsultation using a telehealth station probability.

The aims of experiments 2 and 3 are similar to those of experiment 1, but these experiments address specific subpopulations. Experiment 2 focused on a population composed of elderly people (ie, age >80 years) in nursing homes. This subpopulation is assumed to not be eligible for video TCs and to be medically transported whenever a CS is needed. Based on the observed distances in our data, we assume that nursing homes are located 13.06 km from the clinic. The primary aim of this experiment was to determine whether the use of telehealth stations is more or less efficient for this subpopulation, considering that these individuals are on average located closer to the clinic but cannot use personal transportation. Experiment 3 split the population into 3 groups: patients living within 20 km, between 20 km and 50 km, and more than 50 km from the clinic, and it replicated experiment 1 for each subpopulation. The objective of this experiment is to shed light on the performance of video TCs or TCs using telehealth stations depending on whether the clinic is attractive in an urban, peri-urban, or remote area and to determine the associated break-even points.

Finally, we conducted an extra validation experiment by simulating the base scenario. In this scenario, the parameters are calibrated to reflect the behaviors observed in the data (eg, the intensity of use of each TC type). This validation experiment serves as a robustness check by comparing the observed distribution of the population characteristics with the simulated distributions.

## Results

### Economic Evaluation (Cost Analysis)

We initially observed 532 patients who received at least one TC (ie, TC patient group) and 18,559 CS patients. The TC patients were recruited during a 7-month period (ie, 2020 data) versus 19 months for the CS patients (ie, 2019 and 2020 data). When censoring the data to have the same period of follow-up for each patient in the sample, the sample size fell to 424 TC patients and 13,202 CS patients. Ultimately, the matched sample used in the economic evaluation included 404 patients in each group. However, we used the initial (full) data set to calibrate the parameters in the DES model.

**Figure 2:**
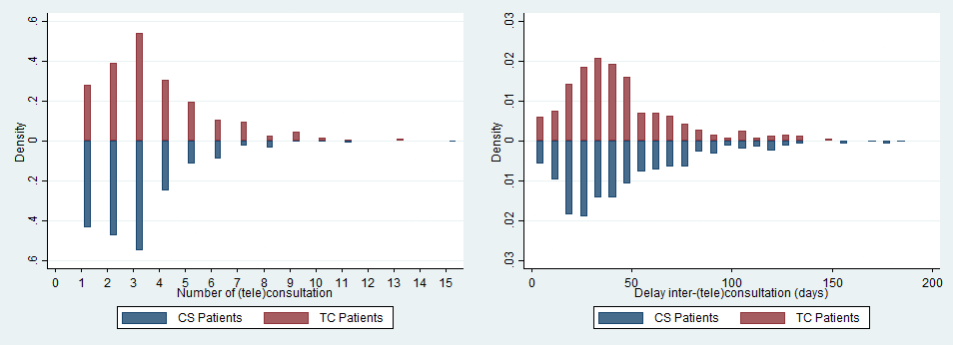
Number of (tele)consultations and time between consultations per patient (matched sample). CS: physical consultation; TC: teleconsultation.

The number of CSs or TCs is likely to have a substantial impact on the total cost per patient. Figure 2 depicts the density of the number of (tele)consultations and the time between consultations per patient for our 2 groups of interest in the matched sample. The average number of (tele)consultations over the period of follow-up per patient was 2.94 for CS patients and 3.53 for TC patients. Interestingly, an unpaired 2-sample *t* test testing the equality of means indicated a significantly higher number of (tele)consultations for TC patients than for CS patients (*P*<.001). Similarly, the average time between consultations for CS patients was 46.5 days, compared with 43.3 days for TC patients. However, an unpaired 2-sample *t* test indicated no significant difference in means (*P*=.17).

As anticipated, we did indeed observe wide differences in individual or disease characteristics between TC and CS patients, which tended to confirm the presence of selection bias (Table 4). For example, Table 4 shows that 49.1% (208/424) of TC patients engaged in (tele)consultations before and after hospitalization, compared with 36.06% (4761/13,202) of CS patients. This distortion in the consultation care pathway might bias the results if left as it stands because it would artificially increase the relative number of (tele)consultations received by TC patients compared with CS patients. The same issue is likely to occur for the wide differences observed between the 2 groups in ICD-10 chapters and care units.

Table 4 shows a good quality of the propensity score matching procedure in balancing the characteristics between our 2 groups of interest. Indeed, while we initially observed wide differences, after matching by the propensity score, there were no longer any differences in ICD-10 chapters, care units, or CS care pathways between TC and CS patients. The matching procedure inherently balanced the sample size of the 2 groups.

A description of the average total cost computed in the matched sample for TC patients and CS patients is provided in Table 5. On average, the total cost per patient over the period of follow-up was €356.37 (US $392) per TC patient and €305.18 (US $336) per CS patient. Interestingly, regarding the composition of the total cost, the cost associated with the tariff of (tele)consultations is higher for TC patients (ie, by €25 [US $28]) than for CS patients. This result confirms the finding in Figure 2, which shows a significantly higher number of (tele)consultations for TC patients (*P*<.001). By contrast, the average cost of transportation per patient is lower for TC patients (ie, by €72.5 [US $80]) than for CS patients. The cost of transportation for TC patients is greater than 0 because 85.4% of TC patients (362/424) had both CSs and TCs during the period of follow-up. Finally, based on the observed TC utilization rate in the study period, the total cost per TC patient includes an additional cost of €98.76 (US $109) for the TC equipment.

**Table 4:** Patient characteristics before and after matching by the propensity score.

Patient characteristics			Before matching by the PS^a^							After 1:1 matching by the PS						
			TC^b^ patients (n=424)		CS^c^ patients (n=13,202)		*P* value^d^		TC patients (n=404)			CS patients (n=404)		*P* value^d^		
Age			61.50		61.48		.98		60.93			60.26		.56		
Female, n (%)			216 (50.94)		7119 (53.92)		.23		207 (51.24)			192 (47.52)		.29		
Male, n (%)			208 (49.06)		6083 (46.08)		.23		197 (48.76)			212 (52.48)		.29		
Travel time to hospital (minutes)			24.25		23.21		.38		24.12			25.32		.56		
Median standard of living (€^e^)			19,765		19,873		.29		19,779			19,921		.33		
Population density			9.49		9.62		.81		9.45			10.49		.35		
**Care pathway consultation, n (%)**																
	After hospitalization	89 (20.99)		3363 (25.47)		.04		70 (17.32)			67 (16.58)		.78			
	Before hospitalization	127 (29.95)		5078 (38.46)		<.01		127 (31.44)			121 (29.95)		.65			
	Both	208 (49.06)		4761 (36.06)		<.01		207 (51.24)			216 (53.47)		.53			
**ICD-10^f^ chapter, n (%)s**																
	Certain infectious and parasitic diseases	0 (0)		51 (0.39)		.21		0 (0)			0 (0)		—^g^			
	Neoplasms	100 (23.58)		1679 (12.72)		<.01		95 (23.51)			91 (22.52)		.74			
	Diseases of the blood and blood-forming organs	0 (0)		15 (0.11)		.51		0 (0)			0 (0)		—			
	Endocrine, nutritional, and metabolic diseases	15 (3.54)		255 (1.93)		.03		14 (3.47)			17 (4.21)		.58			
	Mental disorders	0 (0)		1 (0.01)		.81		0 (0)			0 (0)		—			
	Diseases of the nervous system	1 (0.24)		236 (1.79)		.02		1 (0.25)			2 (0.50)		.56			
	Diseases of the eye	0 (0)		774 (5.86)		<.01		0 (0)			0 (0)		—			
	Diseases of the circulatory system	92 (21.70)		1003 (7.60)		<.01		88 (21.78)			90 (22.28)		.87			
	Diseases of the respiratory system	0 (0)		66 (0.50)		.15		0 (0)			0 (0)		—			
	Diseases of the digestive system	76 (17.92)		2676 (20.27)		.23		72 (17.82)			79 (19.55)		.53			
	Diseases of the skin and subcutaneous tissue	1 (0.24)		157 (1.19)		.08		1 (0.25)			1 (0.25)		.99			
	Diseases of the musculoskeletal system	45 (10.61)		2524 (19.12)		<.01		43 (10.64)			55 (13.61)		.20			
	Diseases of the genitourinary system	55 (12.97)		1106 (8.38)		.001		52 (12.87)			46 (11.39)		.52			
	Pregnancy, childbirth, and the puerperium	0 (0)		1 (0.01)		.86		0 (0)			0 (0)		—			
	Congenital malformations, deformation	2 (0.47)		16 (0.12)		.04		2 (0.50)			1 (0.25)		.56			
	Symptoms not classified elsewhere	10 (2.36)		412 (3.12)		.46		10 (2.48)			7 (1.73)		.46			
	Injury, poisoning	1 (0.24)		286 (2.17)		.008		1 (0.25)			0 (0)		.32			
	Factors influencing health status	26 (6.13)		1944 (14.73)		<.01		25 (6.19)			15 (3.71)		.11			
**Care unit, n (%)**																
	Ambulatory emergency	0 (0)		339 (2.57)		.001		0 (0)			0 (0)		—			
	Ambulatory	143 (33.73)		3427 (25.96)		<.01		143 (35.40)			130 (32.18)		.33			
	Short stay	55 (12.97)		2397 (18.16)		.006		55 (13.61)			58 (14.36)		.76			
	Anesthesia	1 (0.24)		1 (0.01)		<.01		0 (0)			0 (0)		—			
	Other	1 (0.24)		0 (0)		<.01		0 (0)			0 (0)		—			
	Digestive system	56 (13.21)		1539 (11.66)		.33		48 (11.88)			49 (12.13)		.91			
	Sleep assessment	2 (0.47)		974 (7.38)		<.01		2 (0.50)			1 (0.25)		.56			
	Gastroenterology	1 (0.24)		110 (0.83)		.18		1 (0.25)			2 (0.50)		.56			
	Gynecology	23 (5.42)		566 (4.29)		.26		21 (5.20)			14 (3.47)		.23			
	Medicine	4 (0.94)		36 (0.27)		.01		3 (0.74)			2 (0.50)		.65			
	Neurology	7 (1.65)		117 (0.89)		.10		6 (1.50)			5 (1.24)		.76			
	Ophthalmology	0 (0)		22 (0.17)		.39		0 (0)			0 (0)		—			
	Orthopedics	19 (4.48)		1741 (13.19)		<.01		19 (4.70)			31 (7.67)		.08			
	Radiology	0 (0)		1 (0.01)		.86		0 (0)			0 (0)		—			
	Resuscitation	1 (0.24)		193 (1.46)		.04		0 (0)			0 (0)		—			
	Monitoring unit	1 (0.24)		384 (2.91)		.001		1 (0.25)			1 (0.25)		.99			
	Urology	66 (15.57)		712 (5.39)		<.01		61 (15.10)			67 (16.58)		.56			
	Vascular disease	44 (10.38)		643 (4.87)		<.01		44 (10.89)			44 (10.89)		.99			

^a^PS: propensity score.

^b^TC patients: patients having at least one teleconsultation.

^c^CS patients: patients having only physical consultations.

^d^Student *t* test of the difference between TC and CS patients (*P* value); see Multimedia Appendix 2 for a description of the ICD-10 chapters.

^e^€1 = US $1.1 (2020 data).

^f^ICD-10: International Statistical Classification of Disease and Related Health Problems, tenth revision.

^g^No observations.

**Table 5:** Composition of the total cost (matched sample, in euros [2020 data]).

Composition	TC^a^ patients				CS^b^ patients		
	Mean (SD)	Range	Share (%)^c^	Mean (SD)		Range	Share (%)^c^
(Tele)consultation cost	130.25 (75.52)	39.02-478.54	36.76	105.27 (65.9)		35.83-537.45	45.56
Medical transportation (1)	87.91 (99.24)	0-1121.65	19.41	132.33 (410.13)		20.44-6964.79	39.47
Nonmedical transportation (2)	39.45 (65.49)	0-831.13	7.89	67.57 (311.37)		2.05-5272.48	14.97
(1)+(2) Total transportation	127.36 (162.85)	0-1952.78	27.3	199.91 (721.12)		22.49-12237.27	54.44
TC equipment	98.76 (0)	N/A^d^	35.94	N/A		N/A	N/A
Total cost per patient^e^	356.37 (213.58)	137.78-2233.88	100	305.18 (753.53)		58.32-12774.72	100

^a^TC: teleconsultation.

^b^CS: physical consultation.

^c^Share: percentage of the total cost.

^d^N/A: not applicable.

^e^Details about the computation of the total cost per patient are provided in the “Methods” section.

The incremental cost of TCs can thus be computed as follows:



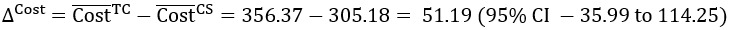



where
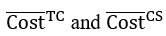
are the average of the total costs per TC patient and CS patient, respectively. Overall, the use of TC increased the total cost per patient by €51.19 (US $56) over the period of follow-up.

The probabilistic sensitivity analysis (ie, nonparametric bootstrapping with 1000 replications) allowed us to compute the 95% CI of the point estimation of the incremental cost. The bootstrap procedure captures a strong uncertainty surrounding the incremental cost estimation, which leads to a wide 95% CI.

**Figure 3:**
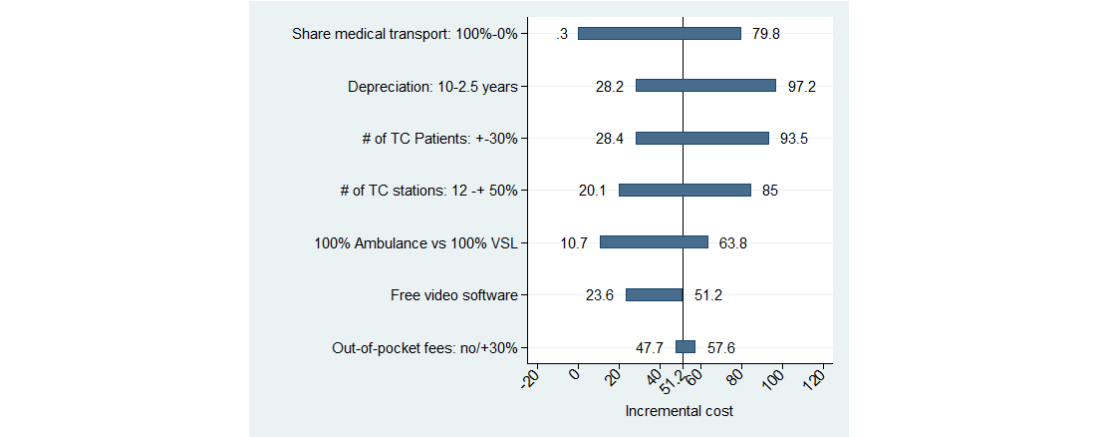
Tornado diagram. TC: teleconsultation; VSL: light health vehicle.

The results of the deterministic sensitivity analysis are presented as a tornado diagram in Figure 3. The share of patients with medical transportation is found to have the largest effect on the incremental cost. Indeed, the use of TCs would be comparable in terms of total cost if 100% of the patients were assumed to be medically transported. Similarly, assuming a 10-year time to full depreciation of TC equipment, the incremental cost would fall to €28 (US $31) per patient. In the tornado diagram, we included a scenario in which the cost of the videoconferencing software would be free to reflect the economic model of some companies. Indeed, to increase TC station sales, some companies were observed to offer the videoconferencing solution for free as a loss leader product. Under this scenario, the incremental cost would decrease on average to €23.6 (US $26).

### Performance Evaluation (DES)

**Figure 4:**
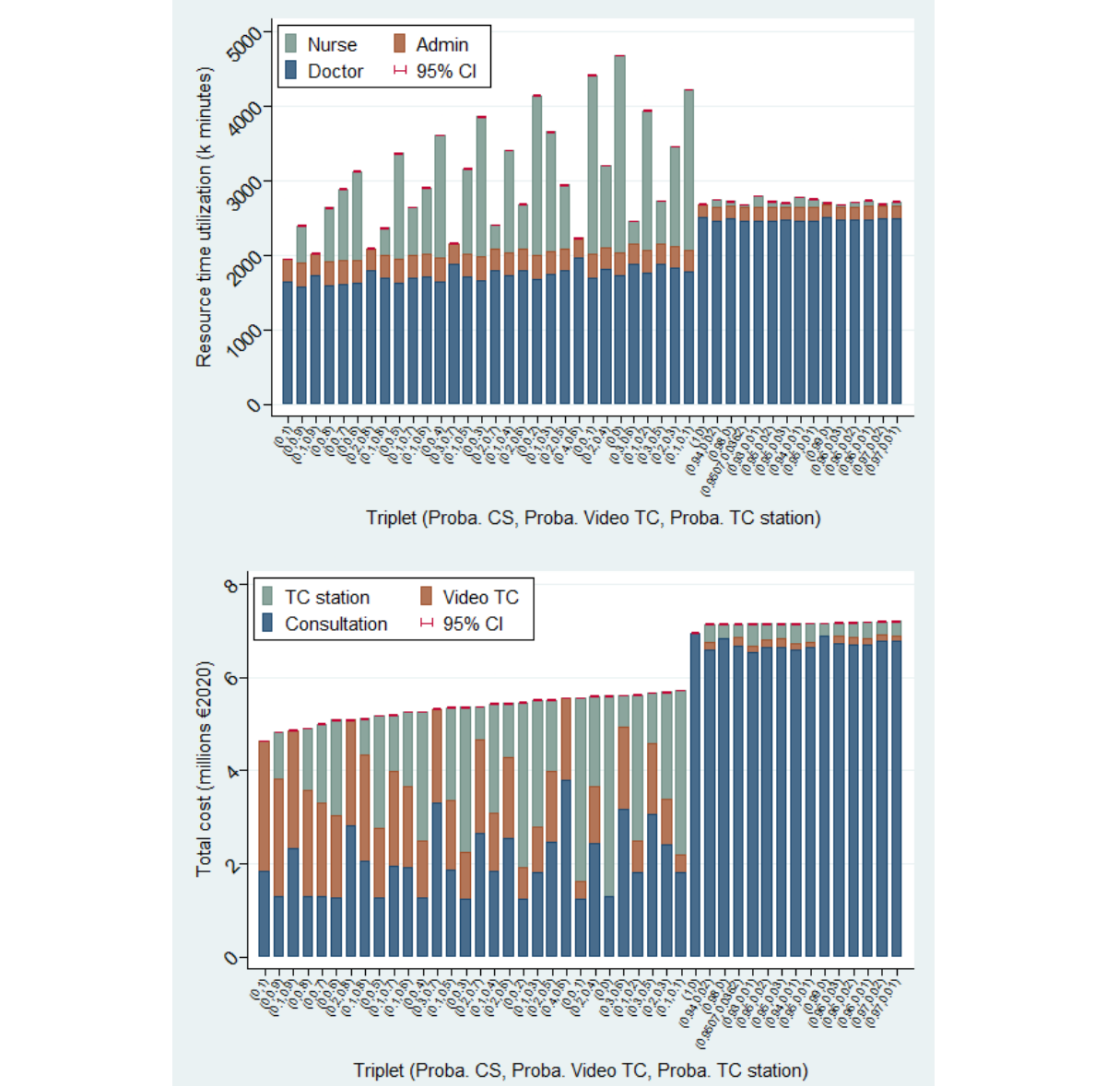
Bar plot of total cost (upper) and resource time utilization (lower) (experiment 1). CS: physical consultation; TC: teleconsultation.

Figure 4 displays the 30 least expensive and the 15 most expensive combinations (*P*^CS^, *P*^visio^, and *P*^TC Station^) in experiment 1 and indicates their total cost (top) and resource consumption (bottom).

Recall that the triplet of probabilities reflects the intensity of use of each type of consultation. For example, (.3,.5) means that *P*^CS^=0.3, *P*^visio^=0.5, and *P*^TC Station^=1 – 0.3 – 0.5=0.2. Using this triplet of probabilities, we simulate an organization of care in which on average, among the population eligible for TC, 30% of consultations are CSs, 50% are video TCs, and 20% are TCs using a telehealth station. Thus, by taking all combinations of this triplet, we simulate a multitude of organizations of care that combine the use of CS, video TC, and TC using a telehealth station (see the “Methods” section for more details). The 14 most expensive scenarios depict situations in which there was an investment in telehealth stations (*P*^TC Station^>0) or a videoconferencing solution (ie, *P*^visio^>0), but in which the intensity of use of TCs remained very low (ie, <1% of the eligible population). Interestingly, the scenario reflecting the intensity of use of each type of consultation observed in the data (*P*^CS^=0.9507, *P*^visio^0.0362, *P*^TC Station^=0.0131) is found to be a more expensive organization of care than a scenario without any TCs (*P*^CS^=1). Comparing the 14 most expensive scenarios and the scenario without any TCs, one can see that the total cost is substantially lowered in the organization of care with a high intensity of use of video TCs or TCs using telehealth stations. The least expensive scenario (*P*^visio^=1) is obtained when all eligible patients are treated through a video TC. Even when *P*^CS^ is set to 0, there is an incompressible volume of CSs because the probabilities (*P*^CS^, *P*^visio^, *P*^TC Station^) are conditional on the eligible population.

**Figure 5:**
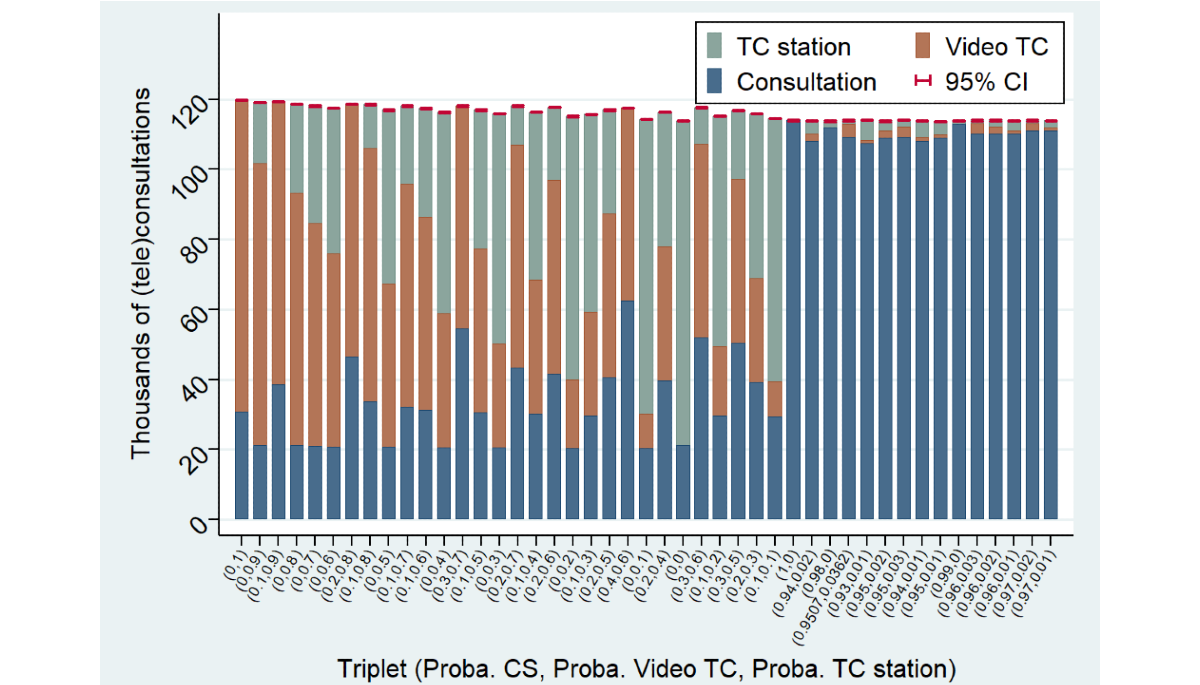
Bar plot of the volume of (tele)consultations for the scenarios used for experiment 1 (same sorting order as Figure 4). CS: physical consultation; TC: teleconsultation.

Regarding this aspect, Figure 5 shows that the incompressible volume of CSs is higher when relying solely on video TCs compared with scenarios mixing the use of video TCs and TCs using telehealth stations. The reason is that elderly people are assumed to be less eligible for video TCs, with a maximum probability *P*^visio^=0.3, as well as because of a positive probability of transfer to a CS at the end of a video TC. Thus, the second least expensive scenario (*P*^CS^=0, *P*^visio^=0.9, *P*^TC Station^=0.1) expands the eligible population and reduces the number of transfers by treating a fraction of the population with TC stations. Additionally, Figure 5 shows that the total number of (tele)consultations completed is slightly higher in scenarios with a higher intensity of video TCs. The reason is that some patients are redirected to a CS after their video TC and due to the lower eligibility of elderly people.

**Figure 6:**
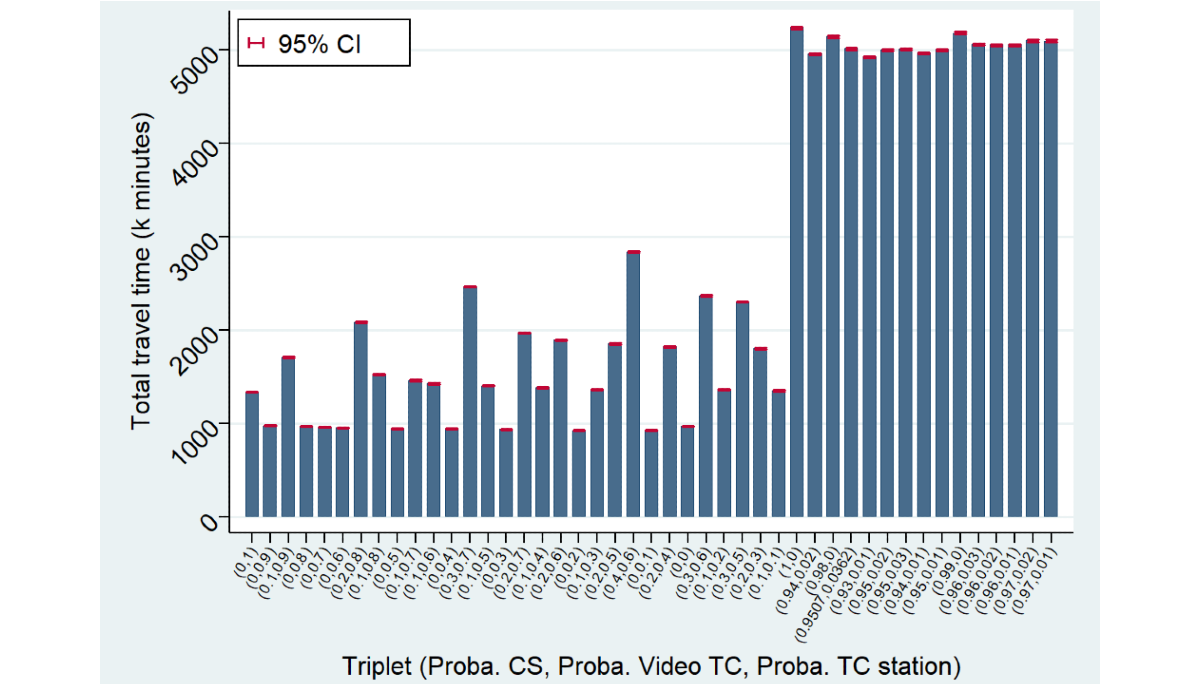
Bar plot of the total travel time (experiment 1). CS: physical consultation; TC: teleconsultation.

However, the sorting of the scenarios based on their total cost has no relationship with their sorting based on the resource time consumption because such consumption is excluded from the total cost computation from a health care sector perspective (Figure 4, bottom). Our results indicate that the use of TCs is time saving for doctors but is time consuming for administrative staff compared with an organization without any TCs. Overall, we found that the use of a videoconferencing solution is time saving because the decrease in doctor time more than compensates for the increase in administrative time. However, scenarios involving the use of telehealth stations also consume nurse time. Taking into account this extra resource, these scenarios tend to be time consuming in terms of total resources and time due to the substantial consumption of nurse time when *P*^TC Station^>0.2, which largely compensates for the associated decrease in doctor time. Ultimately, the scenario relying solely on video TCs (*P*^visio^=1) minimizes the total cost and total resource time consumption while maximizing the total number of (tele)consultations completed for a given population. Nevertheless, relying solely on video TCs does not strictly dominate a combination of video TCs and TCs using telehealth stations because it does not minimize the total travel time KPI (Figure 6). Indeed, the use of TCs (ie, either video TCs or TCs using telehealth stations) naturally leads to substantially lower total travel times compared with CSs. Nevertheless, in this respect, TCs using telehealth stations are even more effective than video TCs. The reason is that a positive share of patients is transferred for CSs after an initial video TC (ie, increased volume) and because of the lower eligibility of elderly people for video TCs, which expands the incompressible volume of CSs.

**Figure 7:**
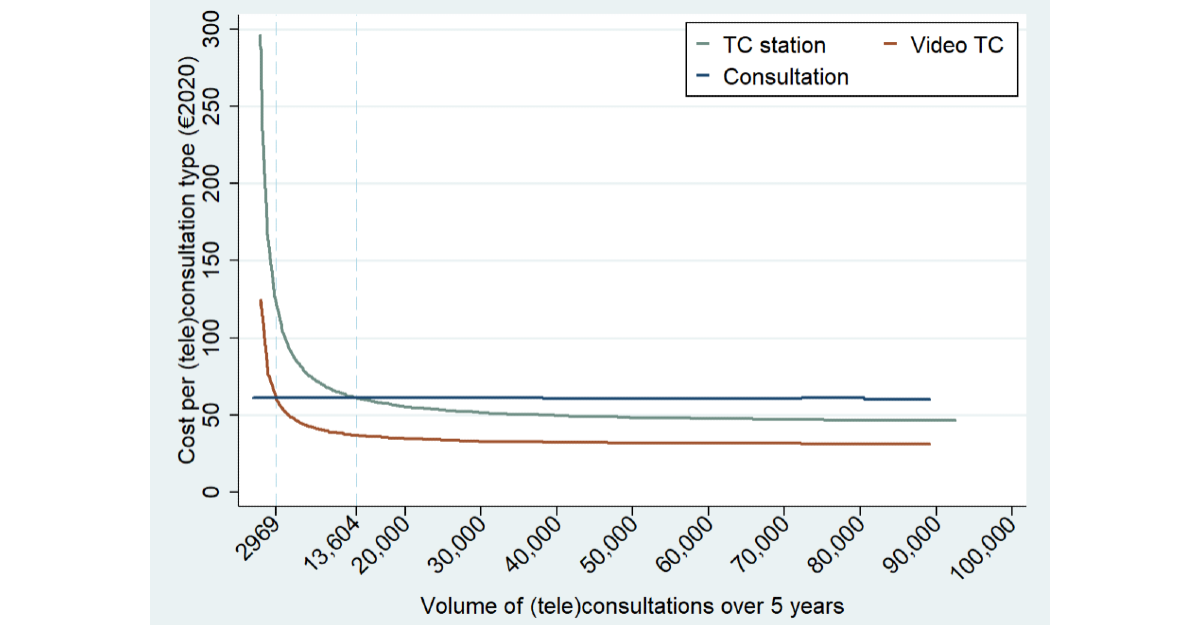
Average total cost (cost per (tele)consultation) for the general population (experiment 1). TC: teleconsultation.

Figure 7 depicts the average cost per (tele)consultation type for a given volume of (tele)consultations completed. The average cost per CS is €60.91 (US $67; range 60.01-60.98) and is broadly constant as there is no fixed investment cost for this consultation type. It only varies with the cost of patient transportation depending on the distances traveled to the hospital by patients in the generated population. Thus, CSs are subject to constant returns to scale. By contrast, the average cost per video TC and per TC using a telehealth station drastically decreases over the number of TCs of each type completed. These inverse function shapes (ie, convex shapes) reflect the initial investment (ie, fixed cost) in a videoconferencing solution or in telehealth stations, which are more or less amortized depending on the number of TCs completed. This implies that the total cost functions for video TCs and TCs using telehealth stations are homogenously of degrees lower than 1. In other words, there are substantial scale economies when investing in a videoconferencing solution or a telehealth station. Thus, the minimum volume required to amortize the videoconferencing solution and telehealth station (ie, with 12 stations) investments is 2969 video TCs and 13,604 TCs using a telehealth station, respectively. At these break-even points, the average cost per CS equals the cost per video TC and per TC using a telehealth station. In other words, the videoconferencing solution is amortized if at least 2.3 video TCs per day are completed on average at the hospital (Table 6). Similarly, a telehealth station is amortized if at least 0.9 TCs per station per day are completed.

**Table 6:** Break-even levels for amortization of TC equipment.

Break levels^a^	Visio TC^b^	12 TC stations	Per TC station
Experiment 1 (population level)	2.3/day	10.8/day	0.9/day
Experiment 3 (subpopulation: urban)	3.5/day	28.3/day	2.4/day
Experiment 3 (subpopulation: peri-urban)	2/day	8.8/day	0.7/day
Experiment 3 (subpopulation: remote)	1.2/day	3.3/day	0.3/day
Experiment 2 (subpopulation: nursing homes)	N/A^c^	2.95/day	0.2/day

^a^The break-even point for the amortization of video TC equipment if we restrict the population to urban agents is 3.5 TCs per day over 5 years (253 working days per year). Computation: 4442 video TCs/1265 working days.

^b^TC: teleconsultation.

^c^N/A: not applicable.

In experiments 2 and 3, we replicated experiment 1 within subpopulations to substantiate the potential variation in amortization speed. The subpopulations included elderly people in nursing homes (experiment 2) and people living in urban, peri-urban, and remote areas (experiment 3). Table 6 displays the break-even points for each subpopulation. The videoconferencing solution is amortized if at least 3.5, 2, and 1.2 video TCs per day are completed for an urban, peri-urban, and remote subpopulation, respectively. Thus, the characteristics of the population that composed the market share of the hospital did indeed have a substantial impact on the amortization speed of the videoconferencing solution. The same pattern can be observed for the amortization of telehealth stations, depending on whether the stations are located in urban, peri-urban, and remote areas, with break-even points of 2.4, 0.7, and 0.3 TCs per station per day, respectively. These results substantiate the importance of the location of telehealth stations, which drastically affects the economic profitability of these stations. Finally, our findings indicate that telehealth stations are even more profitable when they are located in nursing homes, with a break-even point of 0.2 TCs per station per day. The economic profitability of telehealth stations was not as straightforward because nursing homes were located relatively close to the hospital (13.06 km on average), which could have increased the break-even point, as is the case for the urban subpopulation. Nevertheless, the fact that nursing home residents are medically transported whenever physical transportation is needed largely offsets the impact of short distances on the total cost.

Finally, the results of the validation experiment are presented in Multimedia Appendix 4, which shows that our simulation strategy worked well in generating a population with characteristics similar to those of the observed population. Similarly, the share of TCs observed in the data is broadly comparable to the simulated share. The volume of (tele)consultations is naturally substantially higher in the simulation than the observed volumes because we considered a 5-year simulation window against a 7-month period of observations for the 2020 data. We could not consider the transfer KPI or cost KPI in the validation experiment because they were not observed in the data.

## Discussion

### Principal Findings

In this study, we conducted an evaluation of the use of TCs for consultations with specialists at a hospital. Using data on (tele)consultations for any eligible specialty, the key feature of our evaluation framework is the combination of an economic evaluation through a cost analysis and a performance evaluation through a DES approach that distinguishes between 2 types of TCs.

Regarding the cost analysis, the use of TCs was found to increase the total cost per patient by €51.19 (US $56) over a 298-day follow-up (Table 5). Naturally, the fixed cost of investment in a videoconferencing solution and in telehealth stations was one of the main driving factors of the incremental cost. Nevertheless, the incremental cost was also found to be driven by a higher number of (tele)consultations per patient (Figure 2). Thus, our results indicate that during the study period, TCs were used as a complement to CSs rather than as a substitute. A probabilistic sensitivity analysis through a bootstrap procedure indicates a strong uncertainty surrounding the point estimation of the incremental cost. Interestingly, the deterministic sensitivity analysis sheds light on the substantial impact of the share of patients with medical transportation on the incremental cost. Indeed, there would no longer be a difference in total cost per patient if 100% of patients were medically transported whenever a CS was needed (Figure 3). The incremental cost was also found to be sensitive to other cost inputs, such as the volume of TCs completed and the number of TC stations.

The sensitivity of the relative cost of TCs compared with CSs suggests a potential heterogeneity in the profitability of this strategy based on the level of deployment (eg, the volume of TCs completed, the number of telehealth stations) and the characteristics of the population (eg, the share of medical transportation). To delve into this heterogeneity, in this paper, we propose a flexible model for evaluating the performance of various scenarios for future TC deployment using a DES approach. Our findings support the notion that the system obtains the worst performance in terms of total cost in scenarios in which there was an investment in telehealth stations or a videoconferencing solution but in which the intensity of use of TCs remained very low (ie, <1% of the eligible population; Figure 4). The poor performance of these scenarios in terms of costs is induced by the substantial initial investment (ie, fixed costs) in a videoconferencing solution or telehealth stations that is spread over a low volume of TCs. By contrast, scenarios relying intensively on video TCs or TCs using telehealth stations were found to be cost saving compared with the scenario without any TCs owing to the presence of substantial scale economies when investing in TC equipment, while CSs are subject to constant returns to scale. Thus, assuming a 5-year life span of TC equipment (ie, 253 working days/year), the videoconferencing solution and a telehealth station would be amortized (ie, break-even point) if at least 2.3 video TCs and 0.9 TCs using a telehealth station per station were completed each day, respectively (Table 6). Our results also substantiate a strong heterogeneity in the economic profitability of each TC type based on the population characteristics. Indeed, the videoconferencing solution would require a break-even point of 3.5 video TCs per day in an urban population, 2 video TCs per day in a peri-urban population, and 1.2 video TCs per day in a remote population. Thus, the amortization speed when investing in a videoconferencing solution is strongly correlated with the target population of the hospital. Similarly, the location of telehealth stations (ie, relative to the hospital location) drastically affects their break-even points, with daily minimum volumes per station of 2.4, 0.7, and 0.3 for TCs using telehealth stations located in urban, peri-urban, and remote areas, respectively. Interestingly, telehealth stations were found to be even more profitable when located in nursing homes, with a break-even point of 0.2 TCs per day per station. Indeed, even if nursing homes were often located relatively close to the hospital (ie, average distance of 13.06 km), the fact that their residents would need medical transportation whenever a CS was needed drastically reinforced the economic profitability of telehealth stations in this context.

### Limitations

This study also has several potential limitations. We used a retrospective data set to conduct the economic evaluation, and assignment to the treatment and control groups could not be randomized. To overcome this potential risk of selection bias, we conducted 1:1 matching based on the propensity score to derive a pseudorandomized data set. The configuration of our study (ie, a large control group) was suitable for relying on such matching methods, and the matching showed a good performance in balancing the covariates between our 2 groups of interest (Table 4). We could also not distinguish between video TC and TC using telehealth stations at the individual level in the data because we only observed the share of patients using telehealth stations. We thus had to merge these 2 types of TCs into the same group in the economic evaluation and account for this distinction only in the performance evaluation. Similarly, we lacked other individual data, such as data concerning patient transportation mode and the level of out-of-pocket fees for patients, which we replaced by average data at the national level. Another potential limitation concerns the study period, with data on TCs completed in the early stages of the COVID-19 crisis. The fast adoption of telemedicine during that period, as well as the pressure on the health care system, could have had an impact on the behaviors observed in the data. For example, the COVID-19 crisis might have affected the volume of each TC type observed in the data due to the strategy of delayed medical care adopted by many countries to save medical resources [46]. Nevertheless, in this regard, the strength of this study lies in the fact that it is the first to evaluate the use of TCs and the way in which they were used during the study period through an economic evaluation, as well as the fact that this study explores various scenarios for future deployment through a DES approach. Thus, our goal in the DES approach was specifically to explore various levels of intensity of TC use and to assess their relative performance.

### Comparison With Prior Work

In the existing literature, there is no overall consensus regarding the performance of TCs in terms of cost or resource consumption [23,24]. The difficulty of reaching a consensus may partially be explained by the variety of diseases and contexts in which telemedicine has been evaluated. Our results support the notion that the performance of TCs is strongly correlated with the characteristics of the targeted population and the volume of TCs completed. Thus, to reap the full benefit of TC, one needs to identify the most profitable scenarios. In this regard, our results are in line with previous studies and tend to show that the use of TC could be particularly profitable when medical transportation could be avoided [23]. Interestingly, our results also substantiate the claim that telehealth stations could be particularly profitable when located in nursing homes. Indeed, even if nursing homes were located relatively close to the hospital in the case study, the fact that their residents would need medical transportation whenever a CS was needed drastically reinforced the economic profitability of telehealth stations in this context.

Furthermore, while the multitude of diseases investigated independently in a disease-specific setting prevented many studies from considering the amortization of telemedicine equipment, we consider any specialty eligible for TC [6,18-21,23,24]. By so doing, our results substantiate a strong correlation between the break-even levels for TC equipment and the characteristics of the targeted population (ie, their location relative to the hospital). As an illustration, a hospital treating patients living in a radius of 20 km would have to perform 3 times more TCs compared with a hospital treating patients living more than 50 km away to amortize a videoconferencing solution, all else being equal. Similarly, a TC station located close to a hospital (ie, within 20 km) would require 8 times more TCs to be amortized than one located in a remote area (avoiding travel distances >50 km). These findings have important practical implications and substantiate the claim that the strategy of deployment for a TC program should take into account the characteristics of the population and the geographical spread of hospitals and patients within the territory.

Regarding resources other than cost, a recent literature review argued that the use of TCs could provide increased productivity through reduction in consultation time, which might, however, be offset by administrative overhead [23]. In this regard, our results support the notion that the reduction in doctor time resulting from TCs (ie, use of either a videoconferencing solution or a telehealth station) largely offsets the increase in administrative time consumption. Moreover, in the simulation, by distinguishing between video TCs and TCs using telehealth stations, we shed light on another important resource: nurses. When considering nurse time consumption, our findings indicate that the net effect on resource time consumption could result in increased time. However, an increase in resource time consumption is unlikely to affect the total cost from a health care perspective under an activity-based payment scheme. Nevertheless, these variations in resource consumption should be taken into account to calibrate resources properly when introducing a new telehealth station.

### Conclusions

To conclude, the use of TCs has the potential to lead to a major organizational change in the health care system in the near future. Nevertheless, the performance of TCs in terms of cost reduction is strongly related to the context and deployment strategy. Decision makers should, for example, pay attention to the volume of TCs they expect to achieve as well as the characteristics of the targeted population when investing in a TC solution because they have a decisive impact on its economic profitability. Furthermore, while the organizational and economic impacts of TCs are 2 major aspects to be taken into account in future TC development, there are several other important aspects not covered in this study that should be taken into account, such as patients’ satisfaction and access to care as well as adoption by health care professionals.

## Appendix

Multimedia Appendix 1 Optimal period of follow-up.

Multimedia Appendix 2 Description of ICD-10 chapters.

Multimedia Appendix 3 Medical patient transportation cost.

Multimedia Appendix 4 Validation Experiment.

## Abbreviations

CS: physical consultation
DES: discrete-event simulation
DREES: Directorate for Research, Studies, Assessment, and Statistics
EQ-5D-3L: EuroQol 5-dimensional system
ICD-10: International Statistical Classification of Disease and Related Health Problems, tenth revision
ICT: information and communications technology
INSEE: National Institute of Statistics and Economic Studies
KPI: key performance indicator
PS: propensity score
TC: teleconsultation
UML: uniform modeling language
VSL: light health vehicle
WMA: World Medical Association

## References

[1] Weinstein RS, Krupinski EA, Doarn CR (2018). Clinical Examination Component of Telemedicine, Telehealth, mHealth, and Connected Health Medical Practices. Med Clin North Am.

[2] Tedeschi C (2021). Ethical, Legal, and Social Challenges in the Development and Implementation of Disaster Telemedicine. Disaster Med Public Health Prep.

[3] Swinton JJ, Robinson WD, Bischoff RJ (2009). Telehealth and rural depression: physician and patient perspectives. Fam Syst Health.

[4] Nesbitt TS, Cole SL, Pellegrino L, Keast P (2006). Rural outreach in home telehealth: assessing challenges and reviewing successes. Telemed J E Health.

[5] French National Health Insurance (2020). Croissance record du recours à la téléconsultations en mars [Communiqué de presse du 31 mars 2020]. de l'Assurance Maladie.

[6] French National Authority for Health Efficience de la télémédecine : état des lieux de la littérature internationale et cadre d’évaluation (Rapport d’évaluation médico-économique). Haute Autorité de Santé.

[7] European Commission (2018). Market study on telemedecine. European Commission.

[8] NGAP (2020). Nomenclature Générale des Actes Professionnels. NGAP.

[9] French National Health Insurance (2018). Avenant N°15 à la convention nationale du 4 avril 2012 organisant les rapports entre les pharmaciens titulaires d’officine et l’assurance maladie. de l'Assurance Maladie.

[10] Buvik A, Bergmo TS, Bugge E, Smaabrekke A, Wilsgaard T, Olsen JA (2019). Cost-Effectiveness of Telemedicine in Remote Orthopedic Consultations: Randomized Controlled Trial. J Med Internet Res.

[11] Bergmo TS (2010). Economic evaluation in telemedicine - still room for improvement. J Telemed Telecare.

[12] Buvik A, Bugge E, Knutsen G, Småbrekke Arvid, Wilsgaard T (2016). Quality of care for remote orthopaedic consultations using telemedicine: a randomised controlled trial. BMC Health Serv Res.

[13] Flodgren G, Rachas A, Farmer AJ, Inzitari M, Shepperd S (2015). Interactive telemedicine: effects on professional practice and health care outcomes. Cochrane Database Syst Rev.

[14] Buvik A, Bugge E, Knutsen G, Småbrekke A, Wilsgaard T (2018). Patient reported outcomes with remote orthopaedic consultations by telemedicine: A randomised controlled trial. J Telemed Telecare.

[15] Fieux M, Duret S, Bawazeer N, Denoix L, Zaouche S, Tringali S (2020). Téléconsultation en ORL : enquête de satisfaction en période pandémique COVID-19. Annales françaises d'Oto-rhino-laryngologie et de Pathologie Cervico-faciale.

[16] Ramaswamy A, Yu M, Drangsholt S, Ng E, Culligan PJ, Schlegel PN, Hu JC (2020). Patient Satisfaction With Telemedicine During the COVID-19 Pandemic: Retrospective Cohort Study. J Med Internet Res.

[17] Kruse CS, Krowski N, Rodriguez B, Tran L, Vela J, Brooks M (2017). Telehealth and patient satisfaction: a systematic review and narrative analysis. BMJ Open.

[18] Bergmo TS (2009). Can economic evaluation in telemedicine be trusted? A systematic review of the literature. Cost Eff Resour Alloc.

[19] Reardon T (2005). Research findings and strategies for assessing telemedicine costs. Telemed J E Health.

[20] Whitten P, Mair F, Haycox A, May C, Williams T, Hellmich S (2002). Systematic review of cost effectiveness studies of telemedicine interventions. BMJ.

[21] Ekeland AG, Bowes A, Flottorp S (2010). Effectiveness of telemedicine: a systematic review of reviews. Int J Med Inform.

[22] French National Authority for Health (2020). Évaluation économique de la télésurveillance pour éclairer la décision publique. Quels sont les choix efficients au regard de l’analyse de la littérature?. Haute Autorité de Santé.

[23] Snoswell CL, Taylor ML, Comans TA, Smith AC, Gray LC, Caffery LJ (2020). Determining if Telehealth Can Reduce Health System Costs: Scoping Review. J Med Internet Res.

[24] Eze ND, Mateus C, Cravo Oliveira Hashiguchi T (2020). Telemedicine in the OECD: An umbrella review of clinical and cost-effectiveness, patient experience and implementation. PLoS One.

[25] Fanti MP, Mininel S, Ukovich W, Vatta F (2012). Modelling alarm management workflow in healthcare according to IHE framework by coloured Petri Nets. Engineering Applications of Artificial Intelligence.

[26] Dotoli M, Fanti MP, Mangini AM, Ukovich W (2009). A continuous Petri net model for the management and design of emergency cardiology departments. https://reader.elsevier.com/reader/sd/pii/S1474667015307369?token=CFF05A11757AB5114B7E3B85F04EA789B327DE66FDC7B7D46FFA3FB98D9F4F118E213ADF6FE45A48997ABDED3E1B9C31&originRegion=eu-west-1&originCreation=20220509073709.

[27] Dotoli M, Fanti M, Iacobellis G, Martino L, Moretti A, Ukovich W (2010). Modeling and management of a hospital department via Petri nets. https://www.researchgate.net/publication/224128594_Modeling_and_management_of_a_hospital_department_via_Petri_nets.

[28] Hamana S, Augusto V, Xie X (2016). A timed Petri net approach for verification of territorial healthcare information systems.

[29] Lach J, Vázquez R (2004). Simulation model of the telemedicine program. Proceedings of the 2004 Winter Simulation Conference.

[30] Qiao Y, Ran L, Li J (2020). Optimization of Teleconsultation Using Discrete-Event Simulation from a Data-Driven Perspective. Telemed J E Health.

[31] Akuamoah SW, Lu D, Yaro D (2020). Application of Queueing Theory in Dispensation of Resources and Optimization in Teleconsultation. Int. J. Appl. Comput. Math.

[32] Swan B, Shevlin C, Cho A, Phinney D (2018). Simulation tool to evaluate electronic consultations in rheumatology.

[33] Jean C, Jankovic M, Stal-Le Cardinal J, Bocquet J (2017). Predictive modelling of telehealth system deployment. Journal of Simulation.

[34] French National Authority for Health (2020). Guide Méthodologique: Cartographie Des Impacts Organisationnels Pour l’évaluation Des Technologies de Santé. Haute Autorité de Santé.

[35] French National Authority for Health (2020). Methodological Guidance: Choices in Methods for Economic Evaluation. Haute Autorité de Santé.

[36] (2020). JORF n° 0051 du 29 février 2020. Légifrance.

[37] French National Health Insurance (2017). Données statistiques sur la patientèle des professionnels de santé libéraux. de l'Assurance Maladie.

[38] French National Health Insurance (2021). Tarifs conventionnés des ambulances. de l'Assurance Maladie.

[39] French National Health Insurance (2021). Tarifs conventionnés des VSL. de l'Assurance Maladie.

[40] French NHI (2021). Facturation : les taxis conventionnés. de l'Assurance Maladie.

[41] Gonzalez L, Héam J, Mikou M, Ferretti C (2019). Les dépenses de santé en 2018 - édition 2019. Résultats des comptes de la santé. Direction de la Recherche, des Etudes, de l’Evaluation et des Statistiques. Haute Autorité de Santé.

[42] Stuart EA (2010). Matching Methods for Causal Inference: A Review and a Look Forward. Statist. Sci.

[43] Rosenbaum PR, Rubin DB (1983). The central role of the propensity score in observational studies for causal effects. Biometrika.

[44] Efron B (1979). Bootstrap Methods: Another Look at the Jackknife. Ann. Statist.

[45] Gouyon M Consulter un spécialiste libéral à son cabinet : premiers résultats d’une enquête nationale. Études Et Résultats.

[46] Findling MG, Blendon RJ, Benson JM (2020). Delayed Care with Harmful Health Consequences—Reported Experiences from National Surveys During Coronavirus Disease 2019. JAMA Health Forum.

