# Supplementary material for: How We Can Reap the Full Benefit of Teleconsultations: Economic Evaluation Combined With a Performance Evaluation Through a Discrete-Event Simulation
Source: J Med Internet Res. 2022 May 20;24(5):e32002. doi: 10.2196/32002 (PMC9166645; doi:10.2196/32002)

**Appendix A: Optimal period of follow-up.**

When choosing the length of the period of follow-up, there is a trade-off between setting a long period of observation to include the maximum number of (tele)consultations per patient and setting a shorter period to reduce the number of patients dropped at the tail of the study time horizon. We specified the optimal period of follow-up on the basis of the number of TCs excluded from the sample. The rationale behind this rule, instead of choosing the period of follow-up that maximizes the total sample size, is that the number of TCs observed in the sample is drastically lower than the number of CSs. Therefore, the optimal prehospitalization period of follow-up is 165 days, which corresponds to the longest delay between a TC performed before the hospital stay (Figure A1). Thus, all prehospitalization TCs are included in the sample. Similarly, the optimal posthospitalization period of follow-up is 133 days (Figure A2). At this threshold, 62 TCs are excluded from the sample.

Figure A1: Optimal period of follow-up prehospitalization (left) and posthospitalization (right).


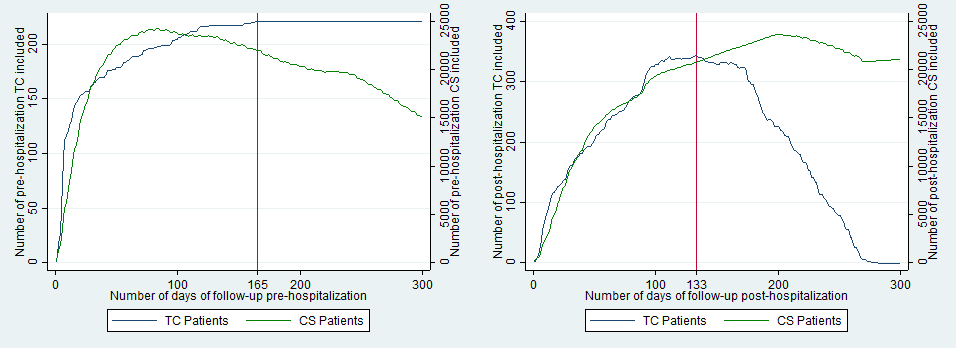

Supplement: Multimedia Appendix 1 [file jmir_v24i5e32002_app1.docx]
