# Supplementary material for: How We Can Reap the Full Benefit of Teleconsultations: Economic Evaluation Combined With a Performance Evaluation Through a Discrete-Event Simulation
Source: J Med Internet Res. 2022 May 20;24(5):e32002. doi: 10.2196/32002 (PMC9166645; doi:10.2196/32002)
Supplement: Multimedia Appendix 2 [file jmir_v24i5e32002_app2.docx]

**Appendix B: Description of ICD-10 chapters**

| **Table B1:** Description of the chapters of the International Statistical Classification of Disease and Related Health Problems 10^th^ revision (ICD-10, V2019 covid expanded) | | |
| --- | --- | --- |
| **Chapter** | **Code group** | **Description** |
| **1** | A00-B99 | Certain infectious and parasitic diseases |
| **2** | C00-D49 | Neoplasms |
| **3** | D50-D89 | Diseases of the blood and blood-forming organs and certain disorders involving the immune mechanism |
| **4** | E00-E89 | Endocrine, nutritional and metabolic diseases |
| **5** | F01-F99 | Mental, behavioral and neurodevelopmental disorders |
| **6** | G00-G99 | Diseases of the nervous system |
| **7** | H00-H59 | Diseases of the eye and adnexa |
| **8** | H60-H95 | Diseases of the ear and mastoid process |
| **9** | I00-I99 | Diseases of the circulatory system |
| **10** | J00-J99 | Diseases of the respiratory system |
| **11** | K00-K95 | Diseases of the digestive system |
| **12** | L00-L99 | Diseases of the skin and subcutaneous tissue |
| **13** | M00-M99 | Diseases of the musculoskeletal system and connective tissue |
| **14** | N00-N99 | Diseases of the genitourinary system |
| **15** | O00-O9A | Pregnancy, childbirth and the puerperium |
| **16** | P00-P96 | Certain conditions originating in the perinatal period |
| **17** | Q00-Q99 | Congenital malformations, deformations and chromosomal abnormalities |
| **18** | R00-R99 | Symptoms, signs and abnormal clinical and laboratory findings, not elsewhere classified |
| **19** | S00-T88 | Injury, poisoning and certain other consequences of external causes |
| **20** | V00-Y99 | External causes of morbidity |
| **21** | Z00-Z99 | Factors influencing health status and contact with health services |
| **22** | U00-U85 | Codes for special purposes |
| **Source:** https://icd.who.int/browse10/2019/en | | |
