# Supplementary material for: How We Can Reap the Full Benefit of Teleconsultations: Economic Evaluation Combined With a Performance Evaluation Through a Discrete-Event Simulation
Source: J Med Internet Res. 2022 May 20;24(5):e32002. doi: 10.2196/32002 (PMC9166645; doi:10.2196/32002)
Supplement: Multimedia Appendix 3 [file jmir_v24i5e32002_app3.docx]

**Appendix C: Medical patient transportation cost**

| **Table C1:** Conventional tariffs for medical patient transportation (in € 2020). Source: French National Health Insurance [38–40]. | | | | |
| --- | --- | --- | --- | --- |
|  | | Light Health Vehicle (VSL) | Ambulance | Taxi |
| Fixed cost | |  |  | 3.5 |
| Waiting cost (per hour) | |  |  | 22.90 |
| Small distance package (up to 8 km) | |  |  | 20 |
| Fixed cost (Zone B) | | 13.45 | 51.3 |  |
| Kilometric rate^a^ | | 0.89 | 2.19 | 0.92 |
| **Surcharge for short distances:** | |  |  |  |
|  | distance ≤ 5 km | 6.26 | 7 |  |
|  | 5 km < distance ≤ 7 km | 6.26 | 5.5 |  |
|  | 7 km < distance ≤ 8 km | 6.05 | 5.5 |  |
|  | 8 km < distance ≤ 9 km | 5.53 | 5.5 |  |
|  | 9 km < distance ≤ 10 km | 5 | 5.5 |  |
|  | 10 km < distance ≤ 11 km | 4.48 | 4 |  |
|  | 11 km < distance ≤ 12 km | 3.96 | 4 |  |
|  | 12 km < distance ≤ 13 km | 3.44 | 4 |  |
|  | 13 km < distance ≤ 14 km | 2.92 | 4 |  |
|  | 14 km < distance ≤ 15 km | 2.4 | 4 |  |
|  | 15 km < distance ≤ 16 km | 1.88 | 2.5 |  |
|  | 16 km < distance ≤ 17 km | 1.36 | 2.5 |  |
|  | 17 km < distance ≤ 18 km | 0.83 | 2.5 |  |
|  | 18 km < distance ≤ 19 km |  | 2.5 |  |
| Probability of use (source: [41]) | | 32% | 22% | 46% |
| Note: kilometer (km); zone B includes the department of Loire (Saint-Etienne); for VSLs and ambulances, the first three kilometers are included in the fixed cost (a). | | | | |
