# Supplementary material for: How We Can Reap the Full Benefit of Teleconsultations: Economic Evaluation Combined With a Performance Evaluation Through a Discrete-Event Simulation
Source: J Med Internet Res. 2022 May 20;24(5):e32002. doi: 10.2196/32002 (PMC9166645; doi:10.2196/32002)
Supplement: Multimedia Appendix 4 [file jmir_v24i5e32002_app4.docx]

**Appendix D: Validation Experiment**

| **Table D1:** Validation experiment comparing the population characteristics in the observed data (observed) with the characteristics of the simulated population in the base scenario (simulated). | | | | | | | |
| --- | --- | --- | --- | --- | --- | --- | --- |
|  | | **Observed** | | **Simulated (20 replications)** | | | |
|  | | **TC** | **CS** | **TC** | **Error** | **CS** | **Error** |
| Age | | 60.91 | 62.23 | 62.73 | 0.44 | 62.3 | 0.09 |
| Female (%) | | 49.49 | 52.26 | 50.24 | 1.46 | 51.16 | 0.31 |
| Travel time (minutes) | | 23.64 | 23.58 | 23.06 | 0.78 | 22.95 | 0.15 |
| **ICD-10 Chapter (%):** | |  |  |  |  |  |  |
|  | Certain infectious and parasitic diseases | 0 | 0.17 | 0.22 | 0.14 | 0.2 | 0.03 |
|  | Neoplasms | 27.36 | 15.69 | 18.59 | 1.14 | 16.21 | 0.21 |
|  | Diseases of the blood and blood-forming organs | 0 | 0.06 | 0 | 0 | 0.08 | 0.02 |
|  | Endocrine, nutritional and metabolic diseases | 4.39 | 2.17 | 2.46 | 0.47 | 2.12 | 0.06 |
|  | Mental disorders | 0 | 0.05 | 0 | 0 | 0.03 | 0.01 |
|  | Diseases of the nervous system | 0.34 | 2.07 | 2.23 | 0.38 | 1.94 | 0.08 |
|  | Diseases of the eye | 0.34 | 7.11 | 8.63 | 0.81 | 7.61 | 0.15 |
|  | Diseases of the circulatory system | 17.57 | 8.6 | 9.82 | 0.84 | 8.44 | 0.23 |
|  | Diseases of the respiratory system | 0 | 0.04 | 0 | 0 | 0.07 | 0.02 |
|  | Diseases of the digestive system | 16.89 | 19.45 | 19.92 | 1.42 | 17.06 | 0.27 |
|  | Diseases of the skin and subcutaneous tissue | 0.51 | 1.03 | 2.02 | 0.45 | 1.71 | 0.07 |
|  | Diseases of the musculoskeletal system | 8.95 | 20.7 | 22.43 | 1.08 | 19.35 | 0.25 |
|  | Diseases of the genitourinary system | 13.01 | 8.04 | 8.63 | 0.86 | 7.45 | 0.16 |
|  | Pregnancy, childbirth and the puerperium | 0 | 0 | 0 | 0 | 0 | 0 |
|  | Congenital malformations, deformation | 0.84 | 0.15 | 0.23 | 0.13 | 0.17 | 0.03 |
|  | Symptoms not classified elsewhere | 2.7 | 1.72 | 2.76 | 0.55 | 2.43 | 0.11 |
|  | Injury, poisoning | 0.17 | 1.34 | 2.06 | 0.45 | 1.73 | 0.08 |
|  | Factors influencing health status | 6.93 | 11.59 | 0 | 0 | 13.4 | 0.21 |
| **Care Unit (%):** | |  |  |  |  |  |  |
|  | - AMBG | 0 | 0 | 0 | 0 | 0 | 0 |
|  | - AMBU | 33.78 | 29.47 | 29.77 | 1.21 | 28.27 | 0.23 |
|  | - AMCC | 11.82 | 21.3 | 20.47 | 0.99 | 18.97 | 0.25 |
|  | - ANES | 0 | 0 | 0 | 0 | 0 | 0 |
|  | - OTHER | 0 | 0 | 0 | 0 | 0 | 0 |
|  | - DIGE | 14.36 | 10.12 | 12.66 | 1.21 | 11.26 | 0.26 |
|  | - EFRS | 1.18 | 5.39 | 0 | 0 | 7.93 | 0.18 |
|  | - GAST | 0.17 | 0.55 | 0.4 | 0.18 | 0.38 | 0.03 |
|  | - GYNE | 5.07 | 5.25 | 5 | 0.91 | 5.17 | 0.13 |
|  | - MEDE | 0.51 | 0.52 | 0.6 | 0.2 | 0.62 | 0.06 |
|  | - NEUR | 1.18 | 1.12 | 0.96 | 0.3 | 0.83 | 0.02 |
|  | - OPHT | 0 | 0.13 | 0 | 0 | 0.14 | 0.02 |
|  | - ORTH | 4.05 | 13.29 | 14.15 | 0.97 | 12.44 | 0.24 |
|  | - RADI | 0 | 0.02 | 0 | 0 | 0.03 | 0.01 |
|  | - REAN | 0 | 0.04 | 0 | 0 | 0.08 | 0.02 |
|  | - SURV | 0.17 | 0.04 | 0.13 | 0.09 | 0.13 | 0.02 |
|  | - UROL | 17.74 | 6.17 | 8.57 | 0.86 | 7.37 | 0.17 |
|  | - VASC | 9.97 | 6.59 | 7.28 | 0.8 | 6.39 | 0.2 |
| Volume of consultation (share) | | 592 (2.79%) | 20643 (97.21%) | 4872.2  (4.27%) | 140.53 | 109045.75  (95.73%) | 659.49 |
| Note: the “Observed” column refers to the 2020 data of the Clinique Mutualiste de Saint-Etienne. The “Simulated” column refers to the simulation run of the base case scenario with 20 replications. Error is computed assuming a normal distribution of the mean over the simulation runs. Video TCs and TCs using telehealth stations are merged into a TC group for purposes of comparison with the observed data. Physical consultation (CS); teleconsultation (TC); ambulatory emergency (AMBG); ambulatory (AMBU); short stay (AMCC); anesthesia (ANES); digestive system (DIGE); sleep assessment (EFRS); gastroenterology (GAST); gynecology (GYNE); medicine (MEDE); neurology (NEUR); ophthalmology (OPHT); orthopedics (ORTH); radiology (RADI); resuscitation (REAN); monitoring unit (SURV); urology (UROL); vascular disease (VASC). See Appendix B for a description of the ICD-10 chapters. | | | | | | | |
